# Prevalence of Oral Mucosal and Extraoral Lesions among Cleft Lip and Cleft Palate Patients Attending to a Private Hospital, India

**DOI:** 10.5005/jp-journals-10005-1075i

**Published:** 2010-09-15

**Authors:** Ajithkrishnan CG, Kiran V Hegde, Sudheer Hongal, Gururaj Patil, Shrinivas Basavaraddi, Thanveer K

**Affiliations:** 1 Professor and Head, Department of Preventive and Community Dentistry, KM Shah Dental College and Hospital, Piparia Vadodara, Gujarat, India; 2 Reader, Department of Pedodontics, KM Shah Dental College and Hospital, Piparia, Vadodara, Gujarat, India; 3 Reader, Department of Preventive and Community Dentistry, People’s Dental Academy, Bhopal, Madhya Pradesh, India; 4 Senior Lecturer, Department of Oral Pathology, Jodhpur Dental College, Jodhpur, Rajasthan, India; 5 Assistant Professor, Department of Orthodontics, SDM Dental College and Hospital, Dharwad, Karnataka, India; 6 Reader, Department of Preventive and Community Dentistry, KM Shah Dental College and Hospital, Piparia, Vadodara Gujarat, India

**Keywords:** Oral mucosal lesions, Extraoral lesions, WHO Proforma 1997, Cleft lip, Cleft palate.

## Abstract

**Aim of the Study :** To assess the prevalence of oral mucosal and extraoral lesions among the cleft lip and/or palate subjects aged between 3 and 18 years, and to compare with those of noncleft controls.

**Methodology :** A cross sectional survey was conducted during the period of March 2004 to September 2004. A select sample of 150 cases consisted of cleft lip and/or palate subjects aged between 3 and 18 years reporting to Outpatient Department, Plastic and Reconstructive Surgery, private Hospital and Medical Research Center, Belgaum. A sample of 450 matched noncleft subjects (Controls) was selected based on convenience from general population. Oral health assessment form as prescribed in Basic Oral Health Surveys, WHO, was used to record the data.

**Results :** Approximately 43% of cleft subjects had abnormalities of upper lip. 7% of clefts and 5% of noncleft controls presented enlarged lymph nodes on palpation in the head and neck region. Both the cleft subjects and noncleft controls had ulcerations in the buccal mucosa. However, there was no significant difference between them. Among few cleft subjects abscess formation was observed in the sulcus region (1.33%) as well as gingiva (2.66%) adjacent to cleft. Similarly among controls also few abscess in sulcus and gingival region were observed.

**Conclusions :** On intra-and extraoral examination of cleft and noncleft subjects for any lesions revealed that clefts as well as noncleft subjects exhibited few intraoral lesions, however clinically or statistically significant differences were not observed.

## INTRODUCTION

Cleft lip/cleft palate is a congenital craniofacial anomaly that has been found in mankind for ages. The earliest evidence of it in antiquity was discovered in an Egyptian mummy. In 1556, Pierse Franco made the earliest record of cleft lip and in 1691, Hendrik Von Roonhuyze made the earliest record of a cleft lip and palate.^1^

Clefts can be caused by a number of factors that affect the expectant mother early in the first trimester of pregnancy. These factors include infection and toxicity, poor diet, hormonal imbalance and genetic interferences.^2^

Depending on its extent, a cleft lip and/palate usually affects other functional areas in a child’s development. Problems may arise pertaining to feeding, facial appearance, speech, hearing, dental functioning and psychosocial development. The affected individual, thus, would require multidisciplinary care from birth until adulthood and perhaps even after.

It has often been speculated that the children with cleft, lip and/or palate have congenital missing teeth, supernumerary teeth and irregular arrangement of teeth that result in inability to maintain a proper oral hygiene and a higher frequency of dental caries.^3^

Cleft lip and/or palate possess a challenge to the health professionals. In order to manage such patients, it is important that one understand the patient’s general conditions, etiology and natural history, complications and prognosis.

There is surprisingly little information on the prevalence of oral mucosal lesions and extraoral lesions among cleft palate and/or lip patients in India. Hence, the present study was planned to investigate the prevalence of oral mucosal lesions and extraoral lesions among cleft palate and/or lip patients reporting to Plastic Surgery Outpatient Department, Private Hospital and Medical Research Center, Belgaum.

## MATERIAL AND METHODS

The present study was conducted to assess the prevalence of oral mucosal lesions and extraoral lesions among the cleft lip and cleft palate patients aged between 3 and 18 years and to compare them with noncleft subjects.

*Source of Data:* Cases consisted of cleft lip and/or palate patients reporting to outpatient department of plastic and reconstructive surgery, Private Hospital and Medical Research Center, Belgaum, Karnataka. A matched control consisting of noncleft subjects was taken from general population in and around Belgaum city.

## METHOD OF COLLECTION OF DATA

### Inclusion Criteria

Patients aged between 3 and 18 years with cleft lip, cleft palate or both belonging to either sex reporting to plastic and reconstructive surgery, Outpatient Department, Private Hospital and Medical Research Center, during the period March 2004 to September 2004 Belgaum, were included.

Control group consisted of subjects aged between 3 to 18 years without cleft lip and/or palate.

Controls were matched with the cases of certain pertinent variables like age, sex, socioeconomic status and geographical distribution.

### Exclusion Criteria

1. Patients with any other facial abnormalities and recognized syndromes.
2. Subjects, who had already undergone complete rehabilitation for cleft lip, cleft palate and alveolus.
3. Subjects with history of any systemic diseases.

A select sample of 150 cases consisted of cleft lip and/ or palate patients. The control group consisted of 450 non-cleft subjects selected based on convenience from general population living in and around Belgaum city, so as to match the study group with respect to certain pertinent variables like age, sex, socioeconomic status and geographic distribution. Ethical clearance was taken from the local Ethical Committee. The clinical examination of all 150 cases and 450 controls constituting the sample was entirely done by only one person. Before conducting the survey, the calibration of examiner was done in order to limit intra-examiner variability. Kappa coefficient was found to be 0.8, reflecting a high degree of conformity in observations. The data of controls was collected at various places in and around Belgaum city by conducting a series of dental camps. The WHO Oral Health Assessment Form (1997) was used for this cross-sectional survey. The format was reproduced from the “Oral Health Surveys-Basic Methods”, 4th Edition, WHO^4^ and was printed. Chi square test was applied wherever applicable. “p” value of less than 0.05 was accepted as indicating statistical significance. Data analysis was carried out using SPSS package (version10.0).

## RESULTS

The age and sex distribution between cleft patients and noncleft controls follow a uniform pattern since the controls are matched with the cases. Cleft as well as controls are stratified based on the age into 4 age groups of 3 to 5 years, 6 to 10 years, 11 to 14 years and 15 to 18 years. Out of 150 cases, a majority, i.e. 43.33% belonged to Group I (clefts of primary palate) and a minority, i.e. 20% belonged to Group II (clefts of secondary palate). 55(36.66%) patients belonged to Group III (combination clefts of primary and secondary palate). 46% of the cleft subjects and 65.11% among noncleft controls used tooth brush with tooth paste/ powder to clean their teeth. 40% of the cleft cases used finger with tooth paste/powder to clean their teeth. This difference was not statistically significant (p > 0.05). None of the subjects brushed twice daily to clean their teeth. The results are depicted in the tables.

## DISCUSSION

A child’s ‘GRIN’ is always a joy forever. To elicit a smile or a giggle from an infant, often one has to move close to its face, make some sound or babble. It is this initial interaction that makes a child feel wanted, and it is perhaps an important step in making the child a social being. A problem like cleft lip, the most common congenital anomaly, distorts a child’s face, sometimes making people turn their heads away.

Even the parents of some such children are reluctant to let others meet them. Shunned by practically everyone, the children are doomed to a life of social isolation and deprivation apart from medical and dental complications.

A cleft lip and cleft palate is caused by the failure of tissues and bones to fuse and close in the foetal stage. Clefts are of three types–an opening in the lip, roof of the mouth (hard palate) and soft tissue at the back of the mouth (soft palate). These openings usually close between 4th and 12th week of pregnancy. But when they fail to close, it results in a cleft lip/cleft palate problem.^5^

Many different etiologic factors have been identified or suggested as the cause of formation of a cleft lip with or without cleft of the palate, as well as cleft palate alone. The causes of clefts are complex and involve both genetic and environmental factors. Such factors include maternal exposure to corticosteroids, retinoic acid, phenytoin, hormonal imbalance and diabetes mellitus. Other teratogenic agents, such as smoking and maternal alcohol exposure have been suggested. Dietary lack of folic acid or ingestion of folic acid antagonists has also been implicated in the formation of clefts in some studies.^6^

A cleft lip/cleft palate problem is the most common birth defect. This problem goes beyond the obvious disfigurement of the face, and extends to shame, stigma, social ostracism and medical and dental impairments that affect speech, hearing, swallowing and teeth formation. A study was planned to assess the orodental status in the cleft affected children. This included the assessment of the prevalence of extraoral lesions, oral mucosal lesions, and furthermore to compare them with matched noncleft controls.

A total of 150 cleft affected cases and 450 noncleft controls were examined according to oral health assessment methodology, WHO 1997.^4^

Extensive exploration of available literature revealed very scarce data related to oral health status among cleft lip and cleft palate subjects. Most of the studies have assessed dental caries, periodontal disease and a very few have assessed oral mucosal lesions. All the studies were discrete and none of them have made any attempt to assess oral and extra mucosal lesions in a comprehensive manner. There are very few studies, which have compared dental caries and periodontal disease among subjects with oral clefts and subjects having no oral clefts.

‘Comparison’ is the most powerful tool in epidemiology. “Asking questions, obtaining the answers, making comparisons and drawing inference” is the tenet of epidemiology. Hence, in the present study, an attempt is made not only to assess the prevalence of oral mucosal lesions and extraoral lesions among cleft subjects but also to compare them with noncleft subjects.

Totally acceptable and valid comparisons could not be done between the present study and already reported studies in the literature due to variations in the study designs, methods and certain other constraints. Nevertheless, a sincere attempt is made to compare wherever possible and to the extent feasible.

### Age and Sex Distribution

In the present study, the cleft affected males (64.67%) were higher compared to females (35.33%) (Table 1). With respect to sex ratio by cleft type, 14 studies (Hixon 1951; Mazaheri 1958; Rank and Thomson 1960; Konx and Braithwaite 1962; Moller 1965; Conway and Wagner 1966; Meskin et al 1968; Chi and Godfrey 1970; Saxen and Lahti 1974; Brogan and Woodings 1974; Tal et al 1974; Saxen 1975; Spry and Nugent 1975; Owens et al 1985) including 5,683 individuals of cleft lip, cleft palate and cleft lip and palate showed that males numbered out females in both cleft lip and cleft lip with cleft palate.^7^ These above studies are similar to our findings. In contrast to our study, Meskin LH and Altemus et al reported that females outnumbered males.^8^

**Table 1:** **Table 1:** Distribution of clefts and controls by age and sex

		*Cleft cases*						*Noncleft controls*					
*Age group*		*Male (%)*		*Female (%)*		*Total*		*Male (%)*		*Female (%)*		*Total*	
3-5 yrs		31 (20.67)		16 (10.67)		47 (31.33)		93 (20.67)		48 (10.67)		141 (31.33)	
6-10 yrs		24 (16)		13 (8.67)		37 (24.67)		72 (16)		39 (8.67)		111 (24.67)	
11-14 yrs		19 (12.67)		13 (8.67)		32 (21.33)		57 (12.67)		39 (8.67)		96 (21.33)	
15-18 yrs		23 (15.33)		11 (7.33)		34 (22.67)		69 (15.33)		33 (7.33)		102 (22.67)	
Total		97 (64.67)		53 (35.33)		150		291 (64.67)		159 (35.33)		450	

The present study shows 43.33% of cases were affected by cleft lip and alveolus, 36% were affected by cleft lip and cleft palate (Table 2). Studies by Hixon (1951), Lowry and Trimble (1977) and Knox and Braithwaite (1962) reported that incidence of cleft lip with palate was greater than those with cleft lip alone and isolated cleft palate among whites (Canada, England, Europe). This overall incidence rate of cleft lip, cleft palate and cleft lip and palate for whites ranged from 0.91 to 2.69 per 1,000.^7^ Stevenson et al (1966), Enganuel et al (1972) in their studies reported that the incidence of cleft lip with cleft palate was greater than that of cleft lip alone and isolated cleft palate among Chinese population.^7^

### Oral Hygiene Practices

In the present study, 96% of the cases and 95.55% of controls brushed their teeth once daily to clean their teeth. None of them among cases and controls brushed twice or more than twice daily. 4% among cases and 4.44% among controls never brushed their teeth (Table 3).

Bian Z et al.^9^ in the study among clefts children reported that 53% brushed once daily while 47% brushed occasionally or never. Dahllof et al^10^ reported that 75% of children with clefts brushed their teeth regularly atleast twice a day and 25% brushed once daily (Table 4). Oral hygiene practices among people do show a lot of variations. It depends upon various factors like social class, literacy status, economic conditions, culture, tradition, beliefs, etc. In our study a majority of the subjects is brushing once daily unlike the studies of Bian Z et al^9^ and Dahllof et al.^10^

**Table 2:** **Table 2:** Percentage distribution of different types of cleft patients

*Groups*		*Types of clefts*				*No. of cases*		*Total*		*Total (%)*	
*Group I		Clefts of		Lip		36 (55.38%)					
		primary palate		Lip and alveolus		29 (44.61%)		65		43.33	
*Group II		Clefts of		Soft palate							
		secondary palate		Soft palate and		11 (36.67%)		30		20	
				Hard palate		19 (63.33%)					
*Group III		Combination of clefts of primary and secondary palate						55		36.66	
		Total						150			

*Kernahan and Stark’s classification (1958) of clefts

**Table 3:** **Table 3:** Distribution of cases and controls by oral hygiene AIDS used

*Agents used*		*Cleft cases*								*Non cleft controls*					
		*M(%)*		*F(%)*		*Total(%)*		*M(%)*		*F(%)*		*Total(%)*		*x^2^* *value*	
Tooth brush/ paste/powder		42.66		52.83		46		65.29		64.77		65.11		*x^2^* = 0.71, p = 0.399 (NS)	
Finger/ tooth paste/ powder		40.20		39.62		40		22.33		25.78		23.55		*x^2^* = 0.22; p = 0.6388 (NS)	
Others		14.33		1.88		10		8.24		4.4		7.11		*x^2^* = 0.85; p = 0.2428 (NS)	
No aids used		3.09		5.66		4		4.23		5.03		4.44			

**Table 4:** **Table 4:** Distribution of cases and controls by frequency of cleaning their teeth

*Frequency of* * brushing*		*Cleft cases*						*Noncleft controls*							
		*M(%)*		*F(%)*		*Total(%)*		*M(%)*		*F(%)*		*Total(%)*		*x^2^ value*	
Once daily		96.9		94.33		96		95.87		94.96		95.55		*x^2^* = 0.01, p = 0.3916 (NS)	
Twice daily/or more		-		-		-		-		-		-				-	
Occasionally		3.09		5.66		4		4.123		5.03		4.44		*x^2^* = 0.00, p = 1.00 (NS)	

### Extraoral Lesions

6% of cases and 3.11% of controls had ulceration in the commisures of lip. This difference was not statistically significant. 7.34% of cases and 5.33% of controls had enlarged lymph nodes in the head and neck region. This could be attributed to untreated or prevailing oral and paraoral infections among cases and controls (Table 5).

### Oral Mucosal Lesions

In the present study, 5.33% of cases and 4.66% of controls had aphthous ulceration in the region of buccal mucosa. 1.33% of cases and 2.66% of controls had abscesses in the region of buccal sulcus. 2.66% of cases and 1.11% of controls had abscess in the region of gingiva. These differences were not significant (Table 6). The abscesses in both cases and controls can be attributed to sequelae of untreated caries and untreated gingival infections.

## CONCLUSIONS

Following are the conclusions of the present study:

1. Cleft lip and/or palate cases had an unpleasant and distorted extraoral appearance due to abnormal lips. Very few cleft and noncleft subjects had abscess formation and ulceration in the buccal mucosa.
2. Handicapped children are more neglected when compared to nonhandicapped children not only by the society but also by the family and the state. Such children are often referred to in the literature as ‘God’s forgotten children. The present study is an attempt to make in roads into one among such handicapped children, who are with oral clefts to obtain the baseline data.
3. These children should be identified as risk groups and required strategies should be developed by the health care services under the support of state and health care programs and should be executed to provide integrated care.

**Table 5:** **Table 5:** Distribution of cases and controls by extraoral lesions

*Sl. no.*		*Extraoral lesions*		*Cases*				*Controls*			
				*No*		*%*		*No*		*%*	
0		Normal extraoral appearance		65		43.33		403		89.56	
1		Ulceration, erosions, fissures (head, neck, limbs)		0		0		0		0	
2		Ulceration,erosion, fissures (nose, cheek, chin)		0		0		4		0.88	
3		Ulceration, erosion, fissures (commisures)		9		6		14		3.11	
4		Ulceration, erosions, fissures (vermillion border)		0		0		0		0	
5		Cancrum oris		0		0		0		0	
6		Abnormalities of upper and lower lips		65		43.33		0		0	
7		Enlarged lymph nodes (head and neck)		11		7.34		24		5.33	
8		Other swellings of face and jaws		0		0		5		1.11	
9		Not recorded		0		0		0		0	
		Total		150				450			

**Table 6:** **Table 6:** Distribution of cases and controls by oral mucosal lesions

*Sl. no.*		*Type of lesion*		*Location*		*Cases*				*Controls*			
						*No.*		*%*		*No.*		*%*	
1.		Malignant tumor		-		0		0		0		0	
2.		Leukoplakia		-		0		0		0		0	
3.		Lichen planus		-		0		0		0		0	
4.		Ulceration, apthous, herpetic, traumatic		Buccal mucosa		8		5.33		21		4.66	
5.		Acute necrotising gingivitis		-		0		0		0		0	
6.		Candidiasis				0		0		0		0	
7.		Abscess		Sulci		2		1.33		12		2.66	
				Alveolar ridges gingiva		4		2.66		5		1.11	
8.		Other conditions		-		0		0		0		0	
